# A retrospective cohort study on the influence of UV index and race/ethnicity on risk of stress and lower limb fractures

**DOI:** 10.1186/1471-2474-14-135

**Published:** 2013-04-12

**Authors:** Scott J Montain, Susan M McGraw, Matthew R Ely, Tyson L Grier, Joseph J Knapik

**Affiliations:** 1 U.S. Army Research Institute of Environmental Medicine, Bldg. 42, Kansas St., Natick, MA, 01760, USA; 2 U.S. Army Institute of Public Health, Aberdeen Proving Ground, MD, USA

**Keywords:** Bone health, Vitamin D, Risk factors, Military

## Abstract

**Background:**

Low vitamin D status increases the risk of stress fractures. As ultraviolet (UV) light is required for vitamin D synthesis, low UV light availability is thought to increase the risk of vitamin D insufficiency and poor bone health. The purpose of this investigation was to determine if individuals with low UV intensity at their home of record (HOR) or those with darker complexions are at increased risk of developing stress fractures and lower limb fractures during U.S. Army Basic Combat Training (BCT).

**Methods:**

This was a retrospective cohort study using the Armed Forces Health Surveillance Center data repository. All Basic trainees were identified from January 1997 to January 2007. Cases were recruits diagnosed with stress fractures and lower limb fractures during BCT. The recruit’s home of record (HOR) was identified from the Defense Manpower Data Center database. The average annual UV intensity at the recruits’ HOR was determined using a U.S National Weather Service database and recruits were stratified into low (≤3.9); moderate (4.0-5.4), and high (≥5.5) UV index regions. Race was determined from self-reports.

**Results:**

The dataset had 421,461 men and 90,141women. Compared to men, women had greater risk of developing stress fractures (odds ratio (OR) = 4.5, 95% confidence interval (95%CI) = 4.4-4.7, p < 0.01). Contrary to the hypothesized effect, male and female recruits from low UV index areas had a slightly lower risk of stress fractures (male OR (low UV/high UV) = 0.92, 95%CI = 0.87-0.97; females OR = 0.89, 95%CI = 0.84-0.95, p < 0.01) and were at similar risk for lower limb fractures (male OR = 0.98, 95%CI = 0.89-1.07; female OR = 0.93, 95%CI = 0.80-1.09) than recruits from high UV index areas. Blacks had lower risk of stress and lower limb fractures than non-blacks, and there was no indication that Blacks from low UV areas were at increased risk for bone injuries.

**Conclusions:**

The UV index at home of record is not associated with stress or lower limb fractures in BCT. These data suggest that UV intensity is not a risk factor for poor bone health in younger American adults.

## Background

Vitamin D is an essential nutrient for bone health. It is obtained primarily by cutaneous synthesis after exposure to sunlight, as well as from dietary sources. The ability and availability of sunlight to stimulate the synthesis of vitamin D is reduced at northern latitudes and more specifically in regions with low ultraviolet radiation [1]. This is particularly true during winter months when the sun angle is lowest and day length is shortest, placing greater necessity on obtaining adequate amounts from food sources. Vitamin D synthesis is also compromised by physically blocking the skin from receiving ultraviolet light with clothing and application of sunscreen. Race/ethnicity potentially exaggerates dependency on food sources of vitamin D for those living in northern latitudes and/or low solar load regions, as dark complexion impairs the ability for low levels of ultraviolet light to penetrate the skin and stimulate vitamin D synthesis [2].

There is growing public health concern that vitamin D deficiency is a contributor to poor health outcomes. Based on either the presence or absence of rickets or a plasma 25-hydroxyvitamin D (25(OH)D) concentration less than 25 nmol/L, there is a high prevalence of vitamin D deficiency in many parts of the world. For example, Koenig and Elmadfa [3] reported that 50% of an Austrian population had plasma 25(OH)D below acceptable levels; with highest incidence in elderly people. Similar findings have been reported for infants and pregnant women in northern or southern latitudes [4-7] and populations living in tropical climates who wear concealing clothing or spend little time out of doors [8,9]. In the United States, the prevalence of low plasma 25(OH)D levels appears to be increasing [10] and the increase in prevalence is affecting all race/ethnicity groups. Ruohola et al. [11] reported that male Finnish Soldiers with a plasma 25(OH)D level below the median value were more likely to develop a stress fracture during training (odds ratio (OR) =3.6; 95% confidence interval (95%CI) = 1.2-11.1). Similarly, Burgi et al. [12] reported that female U.S. navy recruits with 25(OH)D less than 20 ng/ml were nearly twice as likely to develop stress fractures than females with levels greater than 40 ng/ml. Race and ethnicity contribute to relative risk, as Cauley et al. [13] reported that white women with higher plasma 25(OH)D had a lower risk of fracture (OR = 0.55; 95%CI = 0.34-0.89) than white women with low plasma 25(OH)D. However, this was not the case for Black and Asian women, nor Hispanic and American Indian women [13].

Bone fractures remain a prevalent cause of morbidity. In the United States, there are typically 1.5 to 2 million incident fractures annually [14]. In the U.S. military, approximately 2% of service members suffer stress fractures [15], with injury rates for females participating in Basic Combat Training (BCT) 2–10 times higher than men [16]. Marginal calcium and/or vitamin D status may contribute to susceptibility, as dietary calcium and vitamin D supplementation have been associated with a reduced incidence of stress fractures (12.2 vs. 14.6%) in female navy recruits [17]. Although dark complexion inhibits Vitamin D formation, it does not appear to increase the risk of stress fracture per se, as black men and women have had lower incidences of stress fractures than White men and women performing the same military training [16,18]. Lappe et al. [18] reported that 10% and 5% of white and black females developed stress fractures, respectively, during 9-wk of Navy basic training. Similarly, in a cohort of 1,286 women participating in BCT at Ft. Jackson, 3.0% and 1.4% of white and black trainees, respectively, were removed from BCT to recover from stress fractures developed during training (Knapik, Unpublished). Associations between modifiers of vitamin D status, such as latitude (or solar load), season, race/ethnicity and body mass index, and bone health in young adults remain poorly described. A recent evidence-based review on Vitamin D for bone health specifically identified the research need for studies on the influence of latitude, season, ethnicity, and body mass index on vitamin D status and bone health outcomes [14].

The purpose of this investigation was to determine the separate and combined risks of residing in low solar load areas (based on UV index) and race/ethnicity on the likelihood of developing stress fractures and lower limb fractures during participation in Army BCT. It was hypothesized that a) individuals residing in areas with low annualized UV index would have a greater risk of developing stress fractures and lower limb fractures than individuals originating from locations with a high UV index, b) blacks originating from low UV index regions would be at greater risk of developing stress fracture during BCT than blacks originating from high UV regions , and c) white women from areas with a low UV index will be at greater risk of developing stress fractures compared white women from high UV index regions, as well as to other racial/ethnic groups in low and high UV index regions. The product of this work will provide evidence for or against whether the relative risk of bone injury is greater among individuals living in geographic regions where vitamin D synthesis attributable to solar exposure is limited.

## Methods

This retrospective cohort study utilized the Armed Forces Health Surveillance Center (AFHSC) data repository. The study design and methods were reviewed and approved by the Scientific Review Committee and Human Use Review Committee at the US Army Research Institute of Environmental Medicine. The requirement for informed consent was waived as the data received from AFHSC did not have personally identifiable information. Enlisted active duty Soldiers were initially selected from the Defense Manpower Data Center (DMDC) Master Personnel File. Basic trainees were selected by identifying their first demographic record in the DMDC database and must have had a rank E1 to E4 and 17–35 years of age at that first record. DMDC records from January 1997 to January 2007 (11-year period) were searched. In-patient and outpatient medical encounters were obtained from the Standard Ambulatory Data Records (SADR), Standard Inpatient Data Records (SIDR), and the Health Care Service Report/Tricare Encounter Data (HCSR/TED). Medical encounters were requested for the inclusive time from each recruit’s first DMDC record to 10 weeks after the first DMDC record. This time block was selected as it should, in most cases, cover the period of BCT. A sampling period beyond this was not selected because soldiers typically move on to specialized training that likely has variable injury risk depending on the soldiers military occupational specialty (e.g., clerk vs. infantryman).

**Figure 1:**
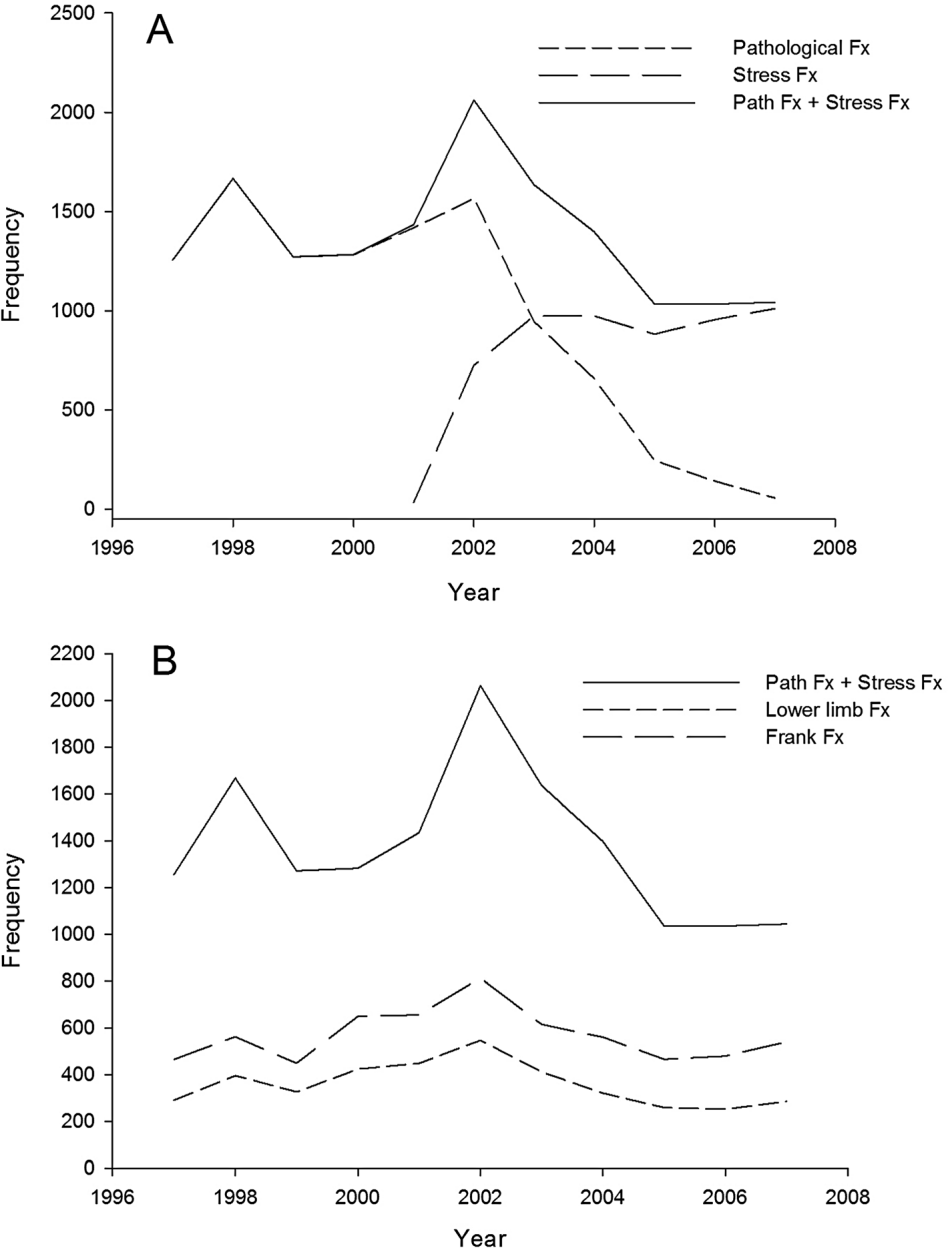
**Bone-related injuries.** Frequency of ICD-9 codes for (**A**) pathological and stress type fractures by year from 1997–2007, and (**B**) pathological + stress fractures, frank fractures, and lower limb fractures from 1997–2007.

The medical encounters were examined to find International Classification of Diseases, Version 9, Clinical Modification (ICD-9-CM) codes for stress fractures, (ICD-9-CM codes 733.1-733.19, 733.93-733.99), lower limb fractures (ICD-9-CM codes 820–829), and all frank fractures (ICD-9 codes 800–829). Cases were Soldiers who were diagnosed with inpatient or outpatient stress fractures, pathological fractures, and frank fractures. Only an individual’s first encounter in any of the 3 categories was considered (i.e., each of the types of injuries was treated as a separate type of case). ICD-9 codes 733.1-733.19 (pathological fractures) were included to identify stress fractures because prior to 2001 these were the codes clinicians in military facilities were instructed to use for stress fractures. It was suspected that some clinicians may have continued to use these codes to record a stress fracture after 2001. It is unlikely that including positive 733.1-733.19 events between 2001 and 2007 confounded the dataset as it is unlikely that BCT will produce many “true” pathological fractures. Figure 1 illustrates the validity of this assumption (Figure 1A) and the year by year frequency of the ICD-9 codes for the various fracture types (Figure 1B).

Also obtained from the AFHSC data repository were DMDC data on demographic information including birth year (for age calculation), educational level, rank, and race/ethnicity. The Military Entrance Processing Station (MEPS) database was used to obtain MEPS location, MEPS zip code, home of record (HOR) state, date of entry into service, sex, height and weight. The UV index of a Soldier’s home prior to entering BCT was determined using their 3 digit HOR zip code.

### Research procedures

The resulting dataset was stratified based on HOR environmental solar load. The annualized UV Index at their HOR was calculated from geographical maps published by the U.S. National Weather Service and Environmental Protection Agency. This was accomplished by dividing the 48 contiguous states and Alaska into 144 cells, calculating the monthly UV index level of each cell, and then calculating the annual average for each cell. The 3-digit zip codes in the database were then aligned to each cell. The validity of this approach was subsequently verified with multiple years of annualized UV Index values for 200 geographically distributed U.S. cities with differing UV index values (source: NOAA, National Weather Service). Our cell values were highly correlated with the city values (r = 0.95) but averaged 0.18 ± 0.28 UV index units lower than the city values. For analysis, three UV index ranges were established: low = less than or equal to 3.9, medium = 4.0-5.4, and high = 5.5 and above. These cut points were chosen as they subdivided the data into 3 approximately equal sized groups. To avoid the possibility that a Soldier changed his HOR upon entry into the military, records from individuals originating from the contiguous US states were only accepted for analysis if both HOR zip code and their Military Entry Processing Station (MEPS) location was in their HOR state or an adjacent state. Exceptions were made, however, for individuals from Hawaii, Alaska and the Virgin Islands, as Soldiers from these areas use MEPS locations outside their HOR state. Records were also removed from consideration if there was a mismatch between the HOR zip code and HOR state.

Sample size estimates were determined using Fisher’s Exact test in nQuery sample size estimating software, with significance level set at alpha = 0.05, power at 80% and a one-tailed test. Based on stress fracture incidence data from a 2007 Army BCT study (n = 4,004; men = 0.56% and women = 2.48%), 645,000 men and 144,000 women would be sufficient to detect a 10% change in incidence rate. Sample size estimates for detecting differences in lower extremity fracture rates (based on incidence rates in the Defense Medical Epidemiological Database of 1% and 1.1% for men and women), 360,000 men and 315,000 women should be sufficient to detect a 10% change in incidence rate whereas 84,000 and 76,500 would be sufficient to detect a 20% change incidence rate.

### Statistical analysis

Descriptive statistics were calculated for all variables. Body mass index (BMI) was calculated as weight/height [2]. Injury incidence for the outcome variables (stress + pathological fractures, lower limb fractures, and frank fractures) were calculated as: recruits with ≥ 1 injury/ total number of recruits in each sex group. Logistic regression was performed to determine the odds ratios (OR) and 95% confidence intervals (95%CI) in order to quantify risks of the outcome measures stratified by gender. Then, HOR UV index was stratified by race/ethnicity and logistic regression was used to assess the relationship of HOR UV index levels for each separate race/ethnicity. IBM SPSS software (version 19) was used to manipulate and analyze the database received from AFHSC.

## Results

The database for analysis was composed of 421,461 men and 90,141 women. Mean ± SD age, height, weight and BMI were 21 ± 3 and 21 ± 4 yr, 176 ± 7 and 163 ± 6 cm, 76.1 ± 13.3 and 61.8 ± 9.5 kg, and 25 ± 4 and 23 ± 3 kg/m^2^ for men and women, respectively. Eighty-nine and 86% of the men and women, respectively, were under 25 years of age. Fifty-five and 67% of men and women, respectively, were in the 18.5-24.9 BMI range. Ninety-two percent were high school graduates; 6% and 8% of men and women, respectively had attended college or possessed a college degree. A variety of race and ethnicities were in the dataset, however, 68% and 51% of the men and women, respectively, reported being White, whereas 15% and 30% of men and women reported being Black. The solar load ranges produced a modest overweighting in the medium UV index range, but 31 and 33% of male data and 35% and 28% of female data were in the high and low UV index groups, respectively.

The incidence of stress fractures, lower limb fractures and frank fractures were 29.5, 7.7 and 12.2 per 1,000 person years, respectively. Table 1 presents the incidence of the outcome measures when stratified by sex. Compared to men, women had a much higher incidence of stress fractures, lower limb fractures, and frank fractures. Univariate analysis indicated that women were 4 times more likely (p < 0.01) to develop a stress fractures than men, 1.7 times more likely (p < 0.01) to develop lower limb fractures, and 1.6 times more likely (p < 0.01) to develop frank fractures.

**Table 1:** Injury incidence (cases/1000 recruits)

	**Men (n = 421,461)**	**Women (n = 90,141)**	**Odds ratio women/men (95% CI)**	**p-value**
Stress fractures	18.8	79.6	4.51 (4.36-4.66)	<0.01
Lower limb fractures	6.9	11.5	1.68 (1.56-1.80)	<0.01
Frank fractures	11.1	17.5	1.59 (1.51-1.69)	<0.01

CI, Confidence Interval.

Low annualized UV index at the HOR did not increase the likelihood of developing stress fractures. The incidence rates for stress fractures were 26.0, 31.3 and 31.1 cases/1,000 person years for low, moderate and high UV index regions, respectively, with those in the low UV index group showing a lower risk compared to the high UV index group (OR low UV/high UV = 0.83, 95%CI = 0.80-0.87; p < 0.01). When stratified by sex, the stress fracture injury risk of men in low UV cohort was lower (p < 0.01) compared to men in high UV group (Table 2). Likewise, women in the low UV group also had a lower injury risk (p < 0.01) compared to women in the high UV index group. Non-Blacks were more likely than Blacks to develop stress fractures during BCT, and the effect magnitude appeared similar between sexes (Table 2).

**Table 2:** Influence of HOR UV index and ethnicity on risk of stress fractures during BCT

		**Men**			**Women**		
**Variable**	**Strata**	**n**	**Odds ratio (95%CI)**	**p-value**	**n**	**Odds ratio (95%CI)**	**p-value**
UV Index	High	130,544	1.0	Referent	31,646	1.0	Referent
	Medium	152,313	1.07 (1.01-1.13)	0.01	33,081	1.02 (0.97-1.08)	0.48
	Low	138,606	0.92 (0.87-0.97)	<0.01	25,414	0.89 (0.84-0.95)	<0.01
Race/ethnicity	White	286,511	1.79 (1.66-1.93)	<0.01	45,779	1.60 (1.51-1.70)	<0.01
	Black	65,270	1.0	Referent	27,261	1.0	Referent
	Hispanic	47,447	1.61 (1.46-1.78)	<0.01	11,570	1.35 (1.24-1.47)	<0.01
	Asian	13,605	1.44 (1.24-1.68)	<0.01	2,897	1.30 (1.12-1.50)	<0.01
	Am. Indian	4,347	1.72 (1.37-2.15)	<0.01	1,513	1.31 (1.08-1.59)	<0.01
	Other	1,183	2.59 (1.84-3.66)	0.01	332	1.74 (1.21-2.50)	<0.01
	Unknown	3,100	1.11 (0.81-1.53)	0.52	789	1.05 (0.78-1.40)	0.77

Am, American.

BCT, Basic Combat Training.

CI, Confidence Interval.

HOR, Home of Record.

UV, Ultraviolet.

Low annualized UV index at HOR also did not increase the likelihood of developing lower limb fractures. The incidence rate was 7.4, 8.0 and 7.8 cases of fracture per 1,000 person years in the low, moderate and high UV index groups, with the odds ratio similar between low and high UV index groups (OR low UV/high UV = 0.95 95% CI = 0.87-1.02; p = 0.17). When stratified by sex, men and women in low UV cohort had similar risk of lower limb fracture than men and women in the high UV group (Table 3). Non-Blacks were more likely than Blacks to develop lower limb fractures (p ≤ 0.01), and similar to stress fractures, the effect magnitude appeared similar between sexes (Table 3).

**Table 3:** Influence of HOR UV index and ethnicity on risk of lower limb fractures during BCT

		**Men**			**Women**		
**Variable**	**Strata**	**n**	**Odds ratio (95%CI)**	**p-value**	**n**	**Odds ratio(95%CI)**	**p-value**
UV Index	High	130,544	1.0	Referent	31,646	1.0	Referent
	Medium	152,321	1.02 (0.94-1.12)	0.63	33,081	1.05 (0.91-1.21)	0.49
	Low	138,605	0.98 (0.89-1.07)	0.66	25,414	0.93 (0.80-1.09)	0.40
Race/ethnicity	White	286,509	2.01 (1.77-2.29)	<0.01	45,779	1.84 (1.57-2.15)	<0.01
	Black	65,270	1.0	Referent	27,261	1.0	Referent
	Hispanic	47,447	1.74 (1.48-2.06)	<0.01	11,570	1.44 (1.15-1.79)	<0.01
	Asian	13,605	1.44 (1.11-1.86)	<0.01	2,897	1.40 (0.96-2.05)	0.08
	Am. Indian	4,347	1.68 (1.14-2.49)	<0.01	1,513	2.00 (1.30-3.08)	<0.01
	Other	1,183	2.88 (1.64-5.04)	<0.01	332	1.58 (0.58-4.27)	0.37
	Unknown	3,100	1.68 (1.07-2.66)	0.03	789	0.66 (0.25-1.78)	0.41

Am, American.

BCT, Basic Combat Training.

CI, Confidence Interval.

HOR, Home of Record.

UV, Ultraviolet.

When the impact of low solar load on stress fractures was examined in each racial group (See Table 4), White men and women from low UV index areas were less likely (p < 0.01) to develop this type of injury when compared to trainees from high UV index areas. The same was true for Asian women. Blacks, Hispanics and American Indians from low UV index areas appeared to be at little different risk of stress fractures than those from high UV index areas. In contrast, Asian men from low UV index regions had a modestly greater risk (p = 0.04) than Asian men from high UV index regions. Similar to low UV index and risk of stress fracture, individuals from moderate UV index areas had similar risk as individuals from high UV index regions; with the exception that Hispanic men from moderate UV index areas were at somewhat greater risk (p < 0.01).

**Table 4:** Impact of HOR UV index on risk of stress fractures when stratified by racial group/ethnicity

		**Men**			**Women**		
**Variable**	**Strata –UV Index**	**n**	**Odds ratio (95%CI)**	**p-value**	**n**	**Odds ratio (95%CI)**	**p-value**
White	High	71,845	1.0	Referent	12,721	1.0	Referent
	Medium	107,128	1.00 (0.93-1.06)	0.87	16,868	0.94 (0.87-1.01)	0.10
	Low	107,536	0.79 (0.74-0.84)	<0.01	16,190	0.74 (0.68-0.80)	<0.01
Black	High	21,196	1.0	Referent	9,888	1.0	Referent
	Medium	29,301	1.12 (0.94-1.32)	0.20	12,124	1.12 (1.00-1.25)	0.05
	Low	14,773	1.09 (0.90-1.33)	0.38	5,249	1.05 (0.91-1.21)	0.52
Hispanic	High	28,724	1.0	Referent	6,725	1.0	Referent
	Medium	9,032	1.35 (1.15-1.60)	<0.01	2,391	1.11 (0.93-1.31)	0.24
	Low	9,691	1.18 (0.99-1.39)	0.06	2,454	1.09 (0.92-1.29)	0.33
Asian	High	5,775	1.0	Referent	1,262	1.0	Referent
	Medium	4,137	1.10 (0.80-1.52)	0.56	915	0.85 (0.62-1.16)	0.31
	Low	3,693	1.39 (1.01-1.90)	0.04	720	0.57 (0.39-0.84)	<0.01
Am. Indian	High	1,331	1.0	Referent	638	1.0	Referent
	Medium	1,530	1.03 (0.62-1.71)	0.92	424	1.32 (0.86-2.04)	0.21
	Low	1,486	0.80 (0.46-1.37)	0.41	451	0.71 (0.43-1.16)	0.17

Am, American.

CI, Confidence Interval.

HOR, Home of Record.

UV, Ultraviolet.

The influence of race/ethnicity on the relationship between solar load and risk for lower limb fracture is presented in Table 5. White men and women from low UV index regions had a lower odds ratio (p < 0.01) for lower limb fracture than those from high UV index areas. Hispanic, Asian and American Indian men and women from low UV index regions had similar risk of lower limb fractures than individuals from high UV index regions. The same was also true for Black women. In contrast, Black men from low UV index regions appeared to be at somewhat increased risk (p = 0.02) of lower limb fracture compared to Black men from high UV index regions. Hispanic women from moderate UV index regions were at elevated risk compared to those from high UV index regions. (p = 0.04).

**Table 5:** Impact of HOR UV index on risk of lower limb fractures stratified by racial group/ethnicity

		**Men**			**Women**		
**Variable**	**Strata –UV index**	**n**	**Odds ratio (95%CI)**	**p-value**	**n**	**Odds ratio (95%CI)**	**p-value**
White	High	71,845	1.0	Referent	12,721	1.0	Referent
	Medium	107,128	0.94 (0.85-1.05)	0.28	16,868	0.94 (0.78-1.13)	0.50
	Low	107,536	0.85 (0.76-0.94)	<0.01	16,190	0.80 (0.65-0.97)	0.02
Black	High	21,196	1.0	Referent	9,888	1.0	Referent
	Medium	29,301	1.33 (0.98-1.80)	0.07	12,124	1.15 (0.85-1.56)	0.37
	Low	14,773	1.48 (1.05-2.09)	0.02	5,249	1.01 (0.68-1.50)	0.97
Hispanic	High	28,724	1.0	Referent	6,725	1.0	Referent
	Medium	9,032	1.26 (0.96-1.67)	0.10	2,391	1.52 (1.01-2.28)	0.04
	Low	9,691	1.11 (0.84-1.47)	0.47	2,454	0.98 (0.61-1.57)	0.94
Asian	High	5,775	1.0	Referent	1,262	1.0	Referent
	Medium	4,137	0.72 (0.40-1.29)	0.27	915	0.92 (0.41-2.05)	0.84
	Low	3,693	1.19 (0.70-2.00)	0.52	720	0.70 (0.27-1.81)	0.46
Am. Indian	High	1,331	1.0	Referent	638	1.0	Referent
	Medium	1,530	1.13 (0.50-2.59)	0.77	424	1.00 (0.41-2.48)	0.99
	Low	1,486	0.45 (0.15-1.31)	0.14	451	0.35 (0.10-1.25)	0.11

Am, American.

CI, Confidence Interval.

HOR, Home of Record.

UV, Ultraviolet.

To assess if the summer months may have masked the effect of low UV index for most of the year, additional analysis was performed that included only trainees beginning military training between September and May. In this subgroup analysis, the stress fracture injury risk of men in the low annualized UV index was similar to the risk in men in the high UV index group (OR low UV/high UV = 0.95, 95%CI = 0.89-1.03; p = 0.20) and females in the low annualized UV index had a modestly lower injury risk compared to women in the high UV index group (OR low UV/high UV = 0.90, 95%CI = 0.83-0.97; p < 0.01). Likewise, a low UV index at HOR did not increase the risk of lower limb fractures during winter months as men in the low UV index group had similar risk of lower limb fracture as men in the high UV index group (OR low UV/high UV = 0.95, 95%CI = 0.84-1.06; p = 0.36), as did women (OR low UV/high UV = 0.93; 95%CI = 0.76-1.15; p = 0.51).

## Discussion

The primary finding of this analysis was that individuals entering military service from areas with a low annualized UV index were actually less likely to suffer a stress fracture than those entering from a high UV intensity area. This observation held true when data from men and women were considered separately or combined. There was no evidence to support the idea that individuals with dark complexion who were residing in low solar load areas were at added risk of developing stress fractures, as individuals self-reporting to be black or of Hispanic ancestry and residing in low UV index areas prior to entering the military had injury risks that were little different from individuals that had been residing in high solar load areas.

The risk of developing a lower limb fracture also appears to be independent of UV intensity at the HOR. Men and women from low UV regions (independent of race/ethnicity) had a similar risk of developing a lower limb fracture compared to those from high UV index regions. When stratified by race/ethnicity, White men and women from low UV index areas actually had a somewhat lower likelihood of developing a lower limb fracture during BCT when compared to those from high UV index regions. While Black men from low UV index regions appeared to have a somewhat elevated risk, this was not apparent in Black women from low UV index areas or Black men from moderate UV index area. Similarly, Hispanic women from medium UV index regions had a modest increase in lower limb fracture risk but this was not evident in men from medium UV index areas or in women from low UV index areas (Table 5). Thus, it is unclear if the modest increase in risk amongst Black men and Hispanic women from low or moderate UV index regions, respectively, reflect true heightened risk, or are just statistical anomalies.

The lack of relationship between UV index and risk of fracture suggests that solar intensity and latitude are not independent risk factors for stress fracture in young men and women. Alternatively, it may be that other risk factors such as socio-economic status, fitness, lifestyle, time outdoors, and body composition mask the effect of UV Index on the risk of lower limb bone pathologies [19-21]. To examine body composition differences between UV index groups, body mass index from their height and weight measures were calculated. Also examined was if there were differences in education levels. In the present data set there were no remarkable differences between UV index groups with respect to the frequency of lower (<18 kg/m2) or higher (≥30 kg/m2) body mass index nor in the incidence of stress fractures at these two BMI extremes. The same was true for education level upon entry into military service (data not shown).

A lack of relationship between latitude and risk of bone injury has been observed in several studies examining lower limb bone fracture incidence amongst older adults [22-26] and female navy recruits [12]. Of particular relevance is the reproducible finding that elderly adults residing in northern regions of the United States have a lower incidence of hip fractures than those residing in the southern part of the country [22,24-26]. The present data, however, contrast with a relatively recent report by Johnell et al. [27] who reported a positive correlation between latitude and probability of hip fracture in older adults. The disparity could be due to differences in experimental approaches and populations studied. To address the relationship between latitude and fracture risk, Johnell et al. searched the existing literature and gathered the incident rates for hip fractures from 35 countries that ranged between 1° and 64° latitude. Johnell et al. data suggest that for each 10° latitude there is a +0.6% increment in 10-year probability that men & women aged 50 yr and above will develop a stress fracture. A close inspection of the data show that 10-yr incidence rates in the 35-40° latitude can vary country to country from 0.3 to 3.9%; moreover, rates in the 3.5-4.0% range occur from 33 to 60° latitude. Therefore, a number of factors independent of latitude appear to contribute to country to country differences in fracture incidence.

Consistent with the literature, the present data indicate that women have a considerably higher risk of stress fractures as well as lower limb fracture and individuals of Black ancestry have less incidence of these pathologies when compared to Whites [18,24]. Also, women entering military basic training were approximately 4 times more likely than men to suffer a stress or pathological fracture, and ~1.7 times more likely to suffer a lower limb fracture. These sex differences in risk compare reasonably well with earlier studies of fracture risk in young individuals participating in military training [16] and older adults [24,28,29]. Similarly, the finding that Black men and women were ~50-80% less likely to develop a lower limb bone injury compares favorably with the ~50% lower risk of stress fractures reported for young adults [18] and for lower limb fractures in elderly adults [24].

A strength of the present experimental approach was the determination of UV index at home of record instead of relying on latitude as the indice of solar load. This is important, as cities/regions of the United States at the same latitude can range from low to high annualized UV index. Moreover, longitudinal differences in cloud cover and elevation have as large an effect on UV intensity as latitude [30]. It can be argued that our stratification into the low, moderate and high UV regions would have been more accurate in terms of ability of the UV light to stimulate vitamin D synthesis, if we had stratified the country using the vitamin D action spectrum weighted UV rather than the UV index. Fioletov et al. [30] have shown that while the UV index reasonably predicts the UV light for vitamin D synthesis in high solar load areas, it overpredicts the availability of UV B light for vitamin D synthesis in low solar load areas. For our analysis, however, the choice of one or the other likely had only modest effects on the stratification as the two indices produce similar UV maps [31].

An assumption of the present study’s approach was that UV index at the home of record would discriminate those with low and high UV exposure and therefore vitamin D status. This in fact may not be the case as vitamin D status is also dependent on time spent out-of-doors, skin exposure, and diet. Regardless, it is reasonable to assume that the lifestyle characteristics of individuals entering the U.S. military would be similar across the United States and with such a large dataset the effect magnitude of UV intensity on bone injury risk should emerge. An *a-priori* criterion for defining a basic trainee, could have also excluded some trainees from the dataset, as some individuals may have had a delayed start date and/or would not have completed BCT in the time period examined. The analysis also relied on ICD-9 codes to tabulate injuries rather than examination of actual medical records. Therefore, it is possible that the incidence of injuries are somewhat over and under reported. A final weakness of this study is that no measures of vitamin D were available. As such, it is unknown if stratifying by UV index sorted the recruits into low, moderate and high vitamin D groups. Future research should identify if there is a relationship between the solar load at home of record and vitamin D status in a cohort of recruits.

## Conclusions

In summary, young adults entering military basic training from regions in the United States with a lower annualized UV index were at no heightened risk of developing stress fractures or lower limb fractures than individuals whose home of record was in medium or high UV index regions. Moreover, when the data were stratified by race/ethnicity, there was no indication that individuals of darker complexion who resided in low UV index regions were at any heightened risk of lower limb bone injury. Taken together, these data suggest that any negative effect of low solar load on vitamin D status and bone health is overridden by other factors that determine resistance to stress-type and lower limb fractures. As a consequence, the UV intensity at home of residence is not predictive of bone injury in younger American adults.

## Competing interests

The authors declare that they have no competing interests.

## Authors’ contributions

SJM was responsible for study design, data analysis, and drafted the manuscript. SMM performed the statistical analysis and aided in data interpretation. MRE assisted with data collection, statistical analysis and manuscript preparation. TLG was responsible for data collection and compilation. JJK assisted in study design, was responsible for data collection and assisted with manuscript preparation. All authors read and approved the manuscript.

## Pre-publication history

The pre-publication history for this paper can be accessed here:

http://www.biomedcentral.com/1471-2474/14/135/prepub
